# Computational biology exposed a common pathogenic mechanism in influenza A and Guillain-Barré syndrome

**DOI:** 10.1371/journal.pone.0349687

**Published:** 2026-05-20

**Authors:** Ye Deng, Zhongwen Tang, Qiong Chen, Xujuan Hu, Yingjie Zhang, Meng Chen, Qiongya Wang, Fuli Ren

**Affiliations:** 1 Clinical Biospecimen Resource Center, Wuhan Jinyintan Hospital, Tongji Medical College of Huazhong University of Science and Technology, Wuhan, Hubei, China; 2 Department of Thoracic Surgery, Wuhan Jinyintan Hospital, Tongji Medical College of Huazhong University of Science and Technology, Wuhan, Hubei, China; 3 Department of Gastroenterology, Wuhan Children’s Hospital, Tongji Medical College of Huazhong University of Science and Technology, Wuhan, Hubei, China; 4 Institute of Aging, Wenzhou Medical University, Wenzhou, China; 5 State Key Laboratory for Diagnosis and Treatment of Severe Zoonotic Infectious Diseases, Wuhan, Hubei, China; Amity University Noida, INDIA

## Abstract

Influenza virus A (H1N1) can lead to acute respiratory infection, while Guillain-Barré Syndrome (GBS) is an autoimmune peripheral neuropathy, which can work as a post-infectious disease. There are clinical observations showing the existence of a possible relationship between the presence of H1N1 infection and GBS disease, and therefore there could be common immunopathological pathways associated with H1N1 infection and GBS. The possible similarity between H1N1 infection and GBS was further investigated using integrated bioinformatics and systems biology approaches. Differentially expressed genes (DEGs) were identified from GEO datasets related to H1N1 infection and GBS. Enrichment analysis was conducted to understand the functional roles of identified DEGs by performing GO and KEGG pathway analysis. For the interacting proteins of the common DEGs, protein-protein interaction (PPI) network analysis helped to find out TLR4, TNF, and ITGAM as key hub genes. These hub genes might have common molecular pathways in H1N1 infection and GBS. To predict possible drug targets for treatment, analysis of interactions between hub genes and miRNAs, TFs, and related diseases was performed.

## Introduction

Seasonal influenza caused by Influenza A leads to seasonal epidemics and, in rare cases, pandemics. It affects millions of people each year and is associated with significant morbidity and mortality rates [1–2]. Among influenza A viruses, the H1N1 subtype is one of the major causes of influenza infections. It can induce the release of cytokines and trigger inflammation by activating both innate and adaptive immune responses, which then leads to clinical manifestations of the disease [3]. GBS is an acute peripheral neuropathy characterized by acute flaccid paralysis. It is thought to occur through molecular mimicry or cross-immune responses that damage the myelin sheath or axons of peripheral nerves [4]. Epidemiological studies have shown that GBS is often preceded by viral or bacterial infections, accounting for approximately 20–30% of cases. Among these infectious triggers, the influenza virus has been identified as a significant inducer of GBS. For example, during the 2009 influenza A (H1N1) pandemic, the number of GBS cases increased markedly [5].

Although clinical observations support a temporal association between influenza and GBS, the molecular mechanism between the two remains unclear. A paucity of research has hitherto been conducted on the common pathogenic network across diseases, with most studies focusing on the immune or genetic characteristics of a single disease. For instance, research has been conducted on the activation of interferon signaling pathways in influenza A or the role of anti-ganglioside antibodies in GBS. Furthermore, extant treatment strategies principally target immune abnormalities in GBS, but prophylactic or targeted interventions for post-influenza A GBS remain limited [6]. In recent years, bioinformatics and systems biology approaches have demonstrated unique advantages in the identification of molecular interaction networks in complex diseases. These approaches include the identification of core regulatory modules through the integration of multi-omics data and the utilization of machine learning models to predict potential therapeutic targets. However, their application to the shared mechanisms linking H1N1 infection and GBS remains limited.

**Fig 1:**
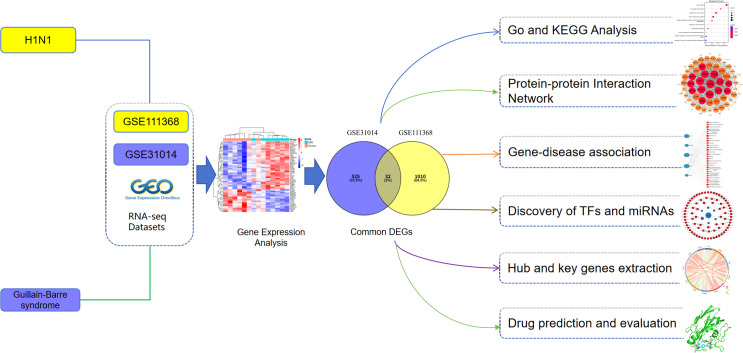
Schematic diagram of the research process in this paper.

We analyzed transcriptome datasets of H1N1 infection and GBS from GEO to identify disease-specific DEGs and their overlap. Given platform and cohort heterogeneity, GO and KEGG enrichment were performed separately for each DEG set, and shared terms/pathways were identified by intersecting significant results. We then constructed an expanded PPI network, prioritized hub genes by network topology, and interrogated regulatory layers via TF/miRNA and gene–disease association networks. Finally, drug repositioning and molecular docking were used to prioritize candidate compounds. The overall workflow is shown in Fig 1.

## Materials and methodss

The GEO database (https://www.ncbi.nlm.nih.gov/geo/; accessed on March 14, 2025) was accessed, and the GSE111368 dataset was selected, which was generated on the GPL10558 microarray platform (Illumina HumanHT-12 V4.0) [7]. The dataset under consideration contains 109 samples infected with H1N1 and 130 samples from healthy subjects. The sequencing platform utilized the Illumina Human HT-12 V4.0 expression microarray chip. The GSE31014 dataset was generated using the GPL96 platform (Affymetrix Human Genome U133A Array [HG-U133A]), comprising seven GBS patient samples and seven healthy control samples [8–9]. All GEO datasets are de-identified (per the ethical standards), and the authors had no access to individual-identifiable information (e.g., names, medical records) during/after data collection. The identification of disease-associated DEGs was achieved through a comparative analysis of the GSE111368 (H1N1 infection) and GSE31014 (GBS) mRNA datasets. Utilizing the limma R package in conjunction with empirical Bayes moderation, we implemented linear models to extract DEGs for each disease. For identifying statistically significant genes, two commonly used thresholds were applied for DEG selection: the adjusted P-value < 0.05, after using the Benjamini–Hochberg correction method and an absolute log2 fold change (|log2FC|) ≥ 1. The adjusted P value criterion was considered to minimize the problem of false discoveries during multiple comparisons, while the second one served to identify differentially expressed genes with both statistical and biological relevance. Finally, shared DEGs between the two datasets were identified via the VENNY v2.1 online tool (https://bioinfogp.cnb.csic.es/tools/venny/) [10–11].

### Functional enrichment analysis of common DEGs

Functional enrichment analysis is a bioinformatics approach that identifies statistically overrepresented biological annotations with the aim of elucidating the potential mechanisms of DEGs. This method identifies significantly enriched GO terms, KEGG pathways, and disease-related signatures. This capability enables the delineation of the functional contributions of specific gene collections to particular pathophysiological processes. In this study, we conducted GO and pathways analyses on H1N1 infection and GBS respectively. We performed GO and KEGG enrichment separately for each dataset and then identified overlapping terms and pathways. Shared items were ranked by adjusted P value, and the top 10 were reported.

Specifically, we used GO analysis to functionally classify common DEGs, including biological processes (BP), molecular function (MF), and cellular components (CC), to clarify their roles in key pathological processes such as immune regulation and neuroinflammation. At the same time, based on KEGG pathway enrichment analysis, the signaling pathways involved in these genes (such as virus-host interaction, autoimmune response, etc.) were further analyzed, to further discover the potential association mechanism between H1N1 infection and the pathogenesis of GBS [11–13].

### Protein-protein interaction network analysis

Proteins execute their mechanistic roles within cellular systems through interactions in PPI networks. To elucidate disease-related protein functions, we constructed PPI networks using common DEGs for subsequent interaction analysis [14]. The analysis was performed using STRING (v12.0; https://www.string-db.org/), a comprehensive database of known and predicted protein associations, including physical interactions and functional linkages. Common DEGs were entered into STRING and a confidence threshold (composite score > 0.4) was applied to generate PPI networks [15–16].

The PPI networks were imported into Cytoscape (v3.10.3) for visualization and downstream analysis. Hub genes were identified using CytoHubba, a Cytoscape plugin that calculates 12 network centrality algorithms to rank node importance [14,17]. Based on validation studies demonstrating its superior discriminative power, the Maximum Clique Centrality (MCC) method was selected to extract the twelve most significant hub genes. To capture key interacting partners, the PPI network was expanded by adding first-shell interactors in STRING, while maintaining the same confidence threshold.

### Transcriptional regulatory network mapping via TF and miRNA interactions

To decode the transcriptional regulatory architecture underlying shared DEGs in H1N1 influenza and GBS, we leveraged the NetworkAnalyst platform (https://www.networkanalyst.ca/) for multi-omics integration [18]. Firstly, we utilized the JASPAR database to identify reliable transcription factor binding sites, and meanwhile combined the experimentally validated miRNA-target gene interactions data in miRTarBase and TarBase [19–21]. We constructed a comprehensive DEG-TF-miRNA regulatory network; subsequently, we used Cytoscape (v3.10.3) to perform network visualization and analysis, focusing on the screening of core transcription factors and key miRNAs that regulate multiple common DEGs. These regulatory nodes may contribute to the shared pathogenesis of the two diseases by influencing virus-host interactions and neuroimmune regulation.

### Gene-disease association analysis networks

The DisGeNET database (https://www.disgenet.org/) was utilized to construct gene-disease association networks, with molecular relationships being systematically aggregated from experimental data, GWAS repositories, and curated literature evidence [22]. The NetworkAnalyst platform was utilized to integrate these associations with multi-omics networks. This facilitated the delineation of disease mechanisms and enabled the construction of comorbidity networks between H1N1 and GBS.

### Therapeutic compound screening via gene signature drug repositioning

We predicted protein-drug interactions (PDIs) and identified repurposable therapeutic agents by targeting common DEGs shared between H1N1 influenza and GBS. Using the Drug Signature Database (DSigDB) embedded within the Enrichr platform, we performed gene set enrichment analysis to discover compounds significantly associated with these DEG signatures (FDR < 0.05) [23]. The Enrichr repository (http://amp.pharm.mssm.edu/Enrichr) provides comprehensive, genome-scale gene set libraries for enrichment analysis, with DSigDB specifically curating drug/compound-target gene relationships accessible through its Disease/Drug Functions module [24].

### Structure-based virtual screening via molecular docking

Molecular docking, a computational drug discovery approach predicting ligand-target binding modes and estimating binding affinities, was used to prioritize candidate therapeutic compounds and explore structure–activity relationships [25]. Based on a systematic literature review, we initially compiled a panel of H1N1-related targets (hemagglutinin/HA, neuraminidase/NA, M2 ion channel, and nucleoprotein/NP) and GBS-associated targets (ganglioside-related components and myelin proteins). To ensure interpretability and consistency with downstream analyses, we focused on NA (a key viral surface enzyme and major drug target) and MPZ (a representative myelin protein implicated in peripheral neuropathy) as the primary targets for detailed docking visualization and reporting, while docking results for other targets were used for preliminary prioritization. High-resolution crystal structures were obtained from the Protein Data Bank (PDB) (via NCBI), and compound structures were retrieved from PubChem. Using the CB-Dock2 platform implementing cavity-detection guided blind docking (https://cadd.labshare.cn/cb-dock2), we performed energy minimization of protein structures with the AMBER force field prior to docking simulations. Binding affinity was quantitatively evaluated by weighted-average Binding Energy (BE) scoring, where BE ≤ −8.0 kcal/mol indicated high-affinity interactions. Finally, binding poses were analyzed in PyMOL v2.5.5 with critical intermolecular distances (H-bond/π-π stacking < 3.5Å) measured, though molecular dynamics simulations were not conducted for conformational validation.

## Results

### Identification of common gene expression signatures between H1N1 and GBS

Severe H1N1 influenza infection has been observed to trigger GBS in susceptible individuals during the post-infection immune dysregulation phase (5 days to 3 weeks) through molecular mimicry mechanisms analogous to Campylobacter jejuni pathogenesis. However, further characterization is required to determine the specific influenza antigenic targets involved in this process.

**Fig 2:**
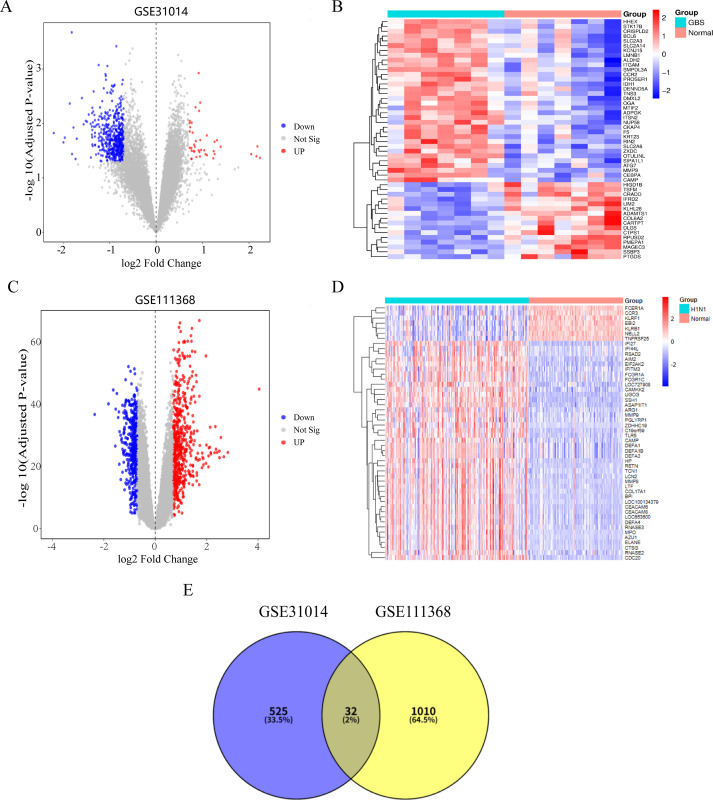
Differential gene expression profiling in GBS and H1N1 influenza datasets. **(A)** Volcano plot of DEGs in GBS dataset (red: up-regulated; blue: down-regulated; gray: non-significant genes; P < 0.05). **(B)** Heatmap clustering analysis of top 50 DEGs in GBS. **(C)** Volcano plot of DEGs in H1N1 dataset. **(D)** Heatmap clustering analysis of top 50 DEGs in H1N1. **(E)** Venny diagram of common DEGs between GSE31014 and GSE111368 datasets.

Transcriptomic profiling of peripheral blood samples (GSE111368 for H1N1 and GSE31014 for GBS) identified 1,043 DEGs in H1N1 (141 upregulated and 902 downregulated) and 559 DEGs in GBS (40 upregulated and 519 downregulated). Volcano plots illustrated prominent expression shifts in both conditions (Figs 2A and 2C), while hierarchical clustering heatmaps revealed distinct disease-specific signatures (Figs 2B and 2D). Of particular significance was the observation that the 12 most significantly dysregulated H1N1-associated DEGs converged on neuroinflammatory pathways and immune response regulation, suggesting shared mechanistic underpinnings. Venny analysis (Fig 2E) delineated 32 conserved DEGs between the two conditions. Functional annotation of the overlapping genes suggested shared biological mechanisms between H1N1 and GBS.

### Functional enrichment analysis of differentially expressed genes

**Fig 3:**
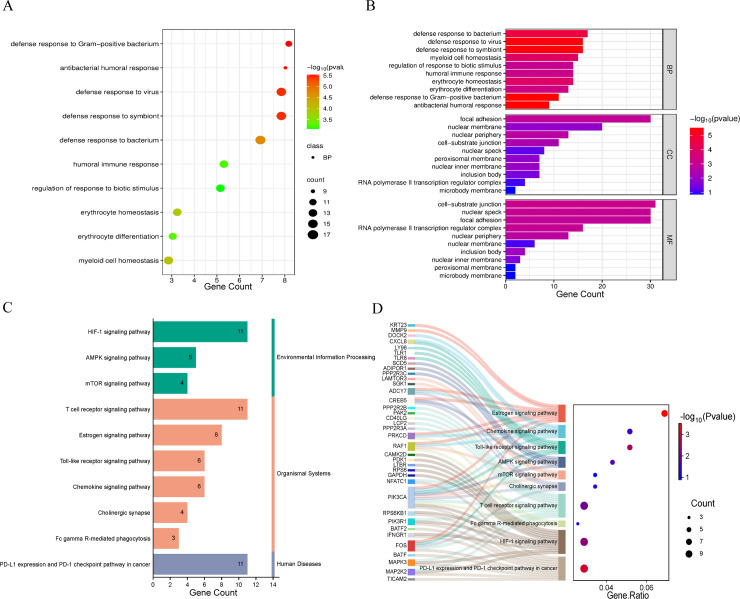
The significance analysis results obtained by analyzing the GO and KEGG evaluations of the two datasets. **(A)** Scatter plot visualization of BP terms significantly enriched by GO analysis. **(B)** The bar chart of GO enrichment analysis divided into BP, CC, MF. **(C)** Bar chart depicting the results of KEGG pathway enrichment analysis for the overlapping pathways identified in the two datasets. **(D)** Sankey diagram visualizing the enrichment relationships of shared KEGG pathways between the two datasets.

Figs 3AB visualize GO enrichment conducted separately on the two dataset-specific DEG lists. Significant terms were then intersected to identify shared functional signals (BP/CC/MF). In the BP category (Fig 3A), host-defense and immune-response terms (e.g., defense response to virus and antibacterial responses) were consistently enriched across datasets, indicating a reproducible immune-activation signature. Fig 3B provides a complementary overview across BP/CC/MF and supports the same shared functional theme.

Figs 3CD summarize KEGG enrichment performed independently for each dataset, followed by intersection of significant pathways (FDR < 0.05). Immune-related pathways and associated regulatory signaling were repeatedly enriched and retained as shared pathways (Fig 3C). The Sankey diagram (Fig 3D) links 37 genes to 10 core pathways, highlighting prominent connections in Toll-like receptor, chemokine, and T-cell receptor signaling and suggesting coordinated immune and metabolic regulation within the shared network.

### Protein interaction network analysis reveals core molecular drivers

**Fig 4:**
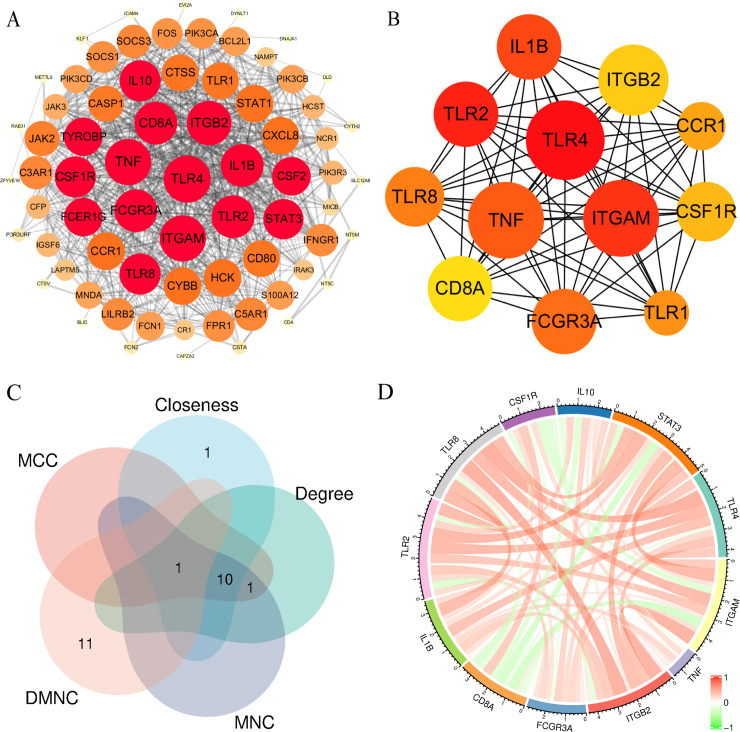
Hub gene identification and interaction analysis. **(A)** PPI network constructed using the 32 shared DEGs as seed genes and expanded by adding first-shell interactors; the resulting network was visualized in Cytoscape. **(B)** Key functional clusters identified through MCC algorithm screening. **(C)** Overlap and concordance of candidate hub genes ranked by five cytoHubba algorithms (Venny diagram). **(D)** Co-expression network of the top 12 MCC-ranked hub genes (as defined from the expanded PPI network).

A PPI network was constructed using the 32 common DEGs that were identified as being differentially expressed between H1N1 influenza and GBS. To capture key interacting partners, the network was expanded by adding first-shell interactors, resulting in a network comprising 73 nodes and 770 edges (Fig 4A). The expanded network was visualized in Cytoscape v3.10.3, with node size and color intensity reflecting interaction centrality. Topological analysis of the expanded PPI network was performed using the cytoHubba plugin with five algorithms (MCC, Degree, MNC, Closeness, and DMNC). Among these, the MCC (Maximal Clique Centrality) algorithm [26] was used to prioritize hub genes because it has been reported to be an effective and sensitive method for identifying essential nodes in biological networks, particularly in PPI network analysis. Based on MCC ranking, the top 12 nodes in the expanded network were defined as core regulatory hub genes for downstream analyses, including TLR4, ITGAM, TNF, ITGB2, FCGR3A, CD8A, IL1B, TLR2, TLR8, CSF1R, IL10, and STAT3 (Table 1). Submodule analysis (Fig 4B) demonstrated tight functional clustering among these hub genes, with TLR4-a pathogen recognition receptor critical for innate immune activation (NCBI Gene: 7099)-emerging as the highest-connectivity node.

**Table 1:** Top-ranked genes identified by cytoHubba using five centrality algorithms (MCC, Closeness, Degree, MNC, and DMNC).

MCC	Closeness	Degree	MNC	DMNC
TLR4	TLR4	TLR4	TLR4	TLR1
ITGAM	TNF	TNF	ITGAM	FPR1
TNF	ITGAM	ITGAM	TNF	LILRB2
ITGB2	ITGB2	ITGB2	ITGB2	MNDA
FCGR3A	IL1B	IL1B	IL1B	CCR1
CD8A	TLR2	TLR2	TLR2	FOS
IL1B	FCGR3A	FCGR3A	FCGR3A	CASP1
TLR2	CD8A	CD8A	CD8A	C5AR1
TLR8	IL10	IL10	IL10	IGSF6
CSF1R	CSF1R	CSF1R	CSF1R	CYBB
IL10	TLR8	TLR8	TLR8	C3AR1
STAT3	STAT3	STAT3	STAT3	TLR2

Further characterization included the creation of a Venny diagram (Fig 4C), which confirmed a high level of concordance between the algorithms in terms of Degree centrality values. Additionally, gene co-expression networks were constructed via gene co-expression network analysis (GCNA) for the top 12 ranked genes (Fig 4D). The integrated results of this study establish that TLR-mediated immune activation and cytokine signaling pathways are central mechanisms that link viral infection and the pathogenesis of autoimmune neuropathy.

### Differential expression analysis of common DEGs between GBS and H1N1

**Fig 5:**
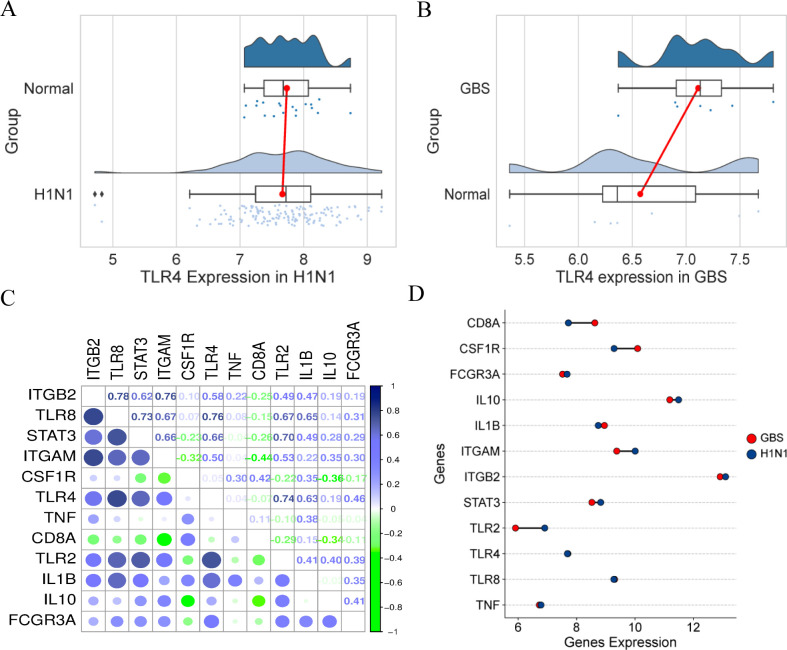
Expression patterns and correlations of key genes in H1N1 and GBS datasets. **(A-B)** Differential expression of TLR4 in H1N1 (A) and GBS (B) datasets. **(C)** Correlation matrix of significantly DEGs. **(D)** Comparative expression profiling of key DEGs across H1N1 and GBS datasets.

The expression profiling of key genes across H1N1 influenza and GBS datasets revealed significant upregulation of TLR4 in GBS samples relative to controls (Figs 5A-B). This finding is consistent with the established role of TLR4 in pathogen recognition via LPS sensing and innate immune activation. A correlation analysis of shared differentially expressed genes was conducted, which identified TLR8 and ITGB2 as exhibiting the strongest positive relationship (r = 0.78, Fig 5C). This finding suggests that TLR8-mediated viral RNA recognition may upregulate ITGB2, thereby enhancing immune cell adhesion and migration during the process of pathogen clearance. The finding is mechanistically reinforced by the co-regulation of TLR4 and TLR8 (r = 0.76), indicating synergistic activation during co-infections that amplifies production of pro-inflammatory cytokines, including TNF-α and IL-6. As illustrated in Fig 5D, ITGB2 demonstrates predominant expression in both disease contexts. In the context of H1N1 infection, this phenomenon may be indicative of viral activation of TLR3/7/8 and RIG-I pathways in myeloid cells, thereby promoting the formation of the ITGB2-integrin complex (CD11b/CD11a), which in turn facilitates the trafficking of immune cells. In contrast, elevated ITGB2 levels in GBS are associated with anti-ganglioside antibody-mediated complement activation. This process involves the recruitment of ITGB2-expressing macrophages and T-cells to peripheral nerves, where they induce demyelination and axonal damage. This correlation has been further substantiated in experimental autoimmune neuritis models, which demonstrate that ITGB2 expression levels are directly proportional to the severity of neuroinflammation. The integrated findings establish a continuum wherein viral-triggered immune mechanisms converge with autoimmune processes through dysregulation of shared molecular pathways involving TLR signaling and integrin-mediated inflammation.

### Transcriptional and post-transcriptional regulatory networks of core H1N1-GBS genes

**Fig 6:**
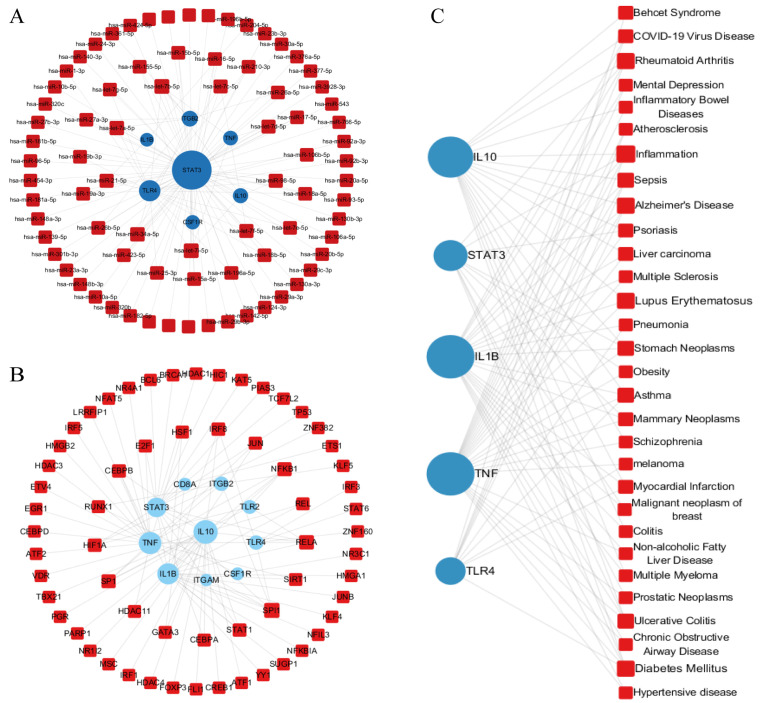
Regulatory networks illustrating interactions among DEGs, miRNAs, TFs, and diseases. **(A)** DEGs-miRNA regulatory network: Blue circular nodes represent DEGs; red square nodes represent miRNAs. **(B)** DEGs-TF interaction network: Blue circular nodes indicate DEGs; red square nodes denote transcription factors. **(C)** Gene-disease association network: Blue circular nodes show DEGs; red square nodes represent disease entities.

In order to elucidate the transcriptional-level regulation of common DEGs, integrated regulatory networks were constructed, mapping TFs and miRNAs through TarBase and miRTarBase databases. As illustrated in Figs 6A-B, the DEG-TF interaction network is visualized, whereby circular nodes represent DEGs and square nodes denote TFs. The size of each node is scaled by its connectivity degree, with larger nodes indicating critical network hubs. Key DEGs with high connectivity (TLR4, STAT3, TNF, ITGAM, IL-10) demonstrate multifaceted roles; a. TLR4 (a key innate immune receptor) detects pathogens and modulates inflammatory responses during H1N1 infection, while its elevated expression in GBS patients correlates with disease severity; b. STAT3 was reported to sustain antiviral signaling despite H1N1 NS1-mediated STAT1 inhibition, thereby maintaining antiviral defenses. Furthermore, the research revealed that STAT3 treatment with STAT3 inhibitors attenuated GBS-like neuroinflammation in experimental autoimmune neuritis (EAN) mice [14]. Among the TFs analyzed, NF-κB, JUN, SP1, GATA3, and HSF1 exhibited the highest regulatory significance.

Complementary networks of DEGs and microRNA (miRNA) (Fig 6C) identified seven genes (STAT3, IL-1β, IL-10, TLR4, TNF, ITGB2, CSF1R) with the strongest interactions between DEGs and miRNAs. The hub miRNAs included hsa-miR-34a-5p, hsa-miR-98-5p, hsa-miR-19a-3p, hsa-miR-27a-3p, and hsa-miR-25a-3p. Notably, hsa-miR-34a-5p has been observed to exhibit dual pathological roles. Firstly, in cases of H1N1, it has been demonstrated to attenuate cytokine storms by inhibiting NF-κB [27]. In GBS, it has been demonstrated that this exacerbates autoimmunity by promoting Th17/M1 polarization, which has been shown to positively correlate with disease severity [28,29]. This suggests that its inhibition could promote neural repair.

### Therapeutic discovery validated by multi-target molecular docking

Our molecular docking strategy targeted pathophysiologically critical proteins: H1N1 surface glycoproteins hemagglutinin (HA) and neuraminidase (NA), which mediate viral-host adhesion [30], alongside GBS autoantigen myelin protein zero (MPZ)–constituting 50–70% of peripheral nerve myelin and triggering demyelination in experimental autoimmune neuritis (EAN) models [31,32]. The details of six potential drug molecules include Parthenolide, Betulinic acid, Tofacitinib, Naringenin, Sulfasalazine and Chelidonine are shown in Table 2. Binding analyses across all prioritized compounds revealed:

**Table 2:** Candidate drugs determined by comprehensive analysis from the DSigDB database (ranked by possibility in the top 6).

High probability associated drugs			
Drugs	Associated direction	Key basis	Compound molecular formula
Parthenolide	H1N1 (Anti-inflammatory), GBS (neuroprotective)	Inhibit the NLRP3 inflammasome and reduce cytokine storms; Neural anti-inflammatory effects were shown in animal models.	C_15_H_20_O_3_
Betulinic Acid	H1N1 (Antiviral/Anti-inflammatory)	Broad-spectrum antiviral (destroying the envelope), inhibiting NF-κB, and possibly alleviating influenza lung injury.	C_30_H_48_O_3_
Tofacitinib Citrate	GBS (Immune Regulation)	JAK3 inhibitors, similar to tofacitinib, can inhibit autoreactive T cells (potentially for autoimmune neuropathy).	C_22_H_28_N_6_O_8_
**Moderate possibility associated drugs**			
**Drugs**	**Associated direction**	**Key basis**	**Compound molecular formula**
Naringenin	H1N1 (Auxiliary anti-inflammatory)	Antioxidant, reduces influenza-associated oxidative stress, but weak direct antiviral effect.	C_15_H_12_O_5_
Sulfasalazine	H1N1 (immunomodulation)	Inhibition of NF-κB, which may alleviate excessive inflammation, but no direct antiviral data.	C_18_H_14_N_4_O_5_S
(+)-Chelidonine	GBS (theoretical neuroprotection)	Anti-inflammatory and microtubule inhibitory effects, but high toxicity and need for structural modification.	C_20_H_19_NO_5_

**Fig 7:**
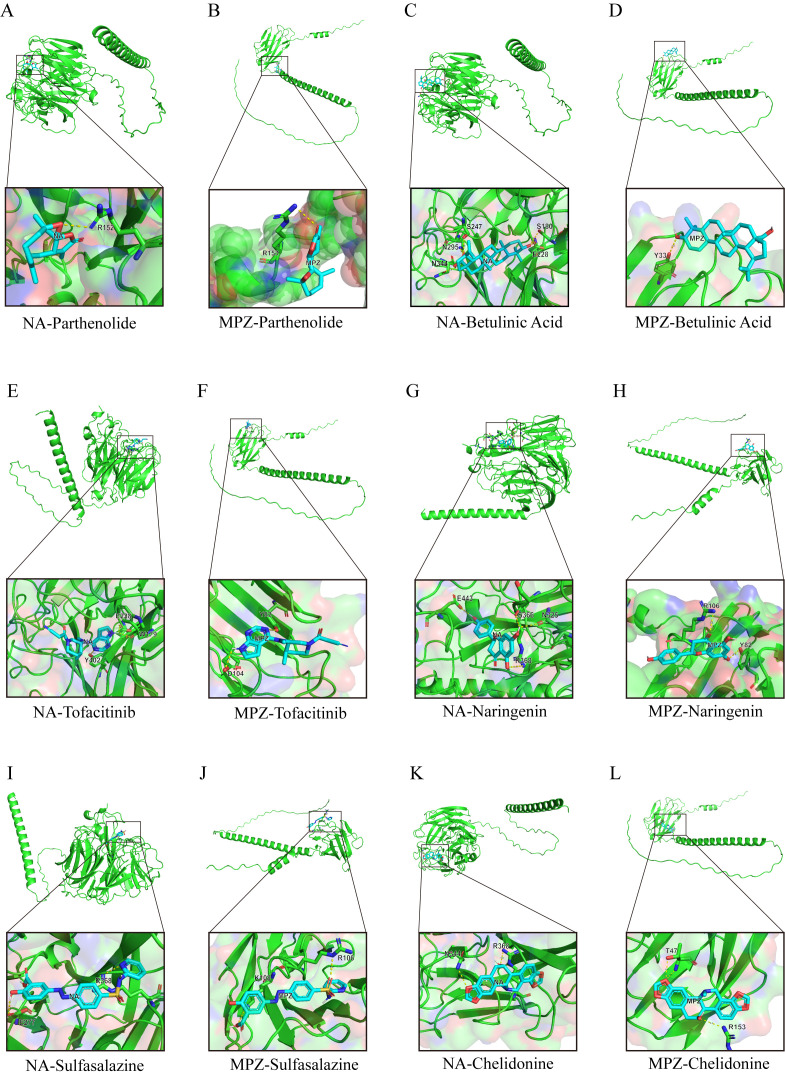
Molecular docking simulations illustrate drug-protein interactions, elucidating binding mechanisms to facilitate drug discovery. Shown are docking models for:(A-B) Parthenolide with H1N1 neuraminidase (NA) and GBS myelin P0 protein (MPZ); **(C-D)** Betulinic Acid with NA and MPZ; **(E-F)** Tofacitinib with NA and MPZ; **(G-H)** Naringenin with NA and MPZ; **(I-J)** Sulfasalazine with NA and MPZ; **(K-L)** Chelidonine with NA and MPZ.

Parthenolide formed hydrogen bonds with NA catalytic residues (Fig 7A), validating its replication inhibition [33], and may exert anti-inflammatory effects via IKK/NF-κB inhibition [34], while hydrophobic interactions stabilized MPZ (Fig 7B), supporting neuroregeneration [35]. Betulinic acid occupied NA’s sialic acid pocket (Fig 7C) via van der Waals forces [36], and anchored MPZ transmembrane domains (Fig 7D) to attenuate neuroinflammation [37].

Tofacitinib established salt bridges with NA (Fig 7E), corroborating JAK-STAT blockade [38], while hydrogen bonding to MPZ extracellular loops (Fig 7F) aligned with immunomodulation [39]. Naringenin formed π-π stacking with NA active sites (Fig 7G) and stabilized MPZ dimers (Fig 7H), indicating flavonoid-mediated membrane stabilization.

Sulfasalazine exhibited π-cation interactions with both NA (Fig 7I) and MPZ (Fig 7J), suggesting novel target engagement. Chelidonine, an isoquinoline alkaloid–demonstrated unanticipated binding to NA catalytic triad (Fig 7K) and MPZ immunoglobulin domains (Fig 7L), warranting further exploration.

## Discussion

Immunopathologic associations between influenza A (H1N1) infection and GBS have also been predicted by our network analysis and hub genes identified by it. This may include shared inflammatory and immunomodulatory pathways. For example, the TLR4-mediated signaling pathway could increase the production of inflammatory cytokines (IL-6 and TNF-α) via the mediators such as STAT3 and NF-κB. Previous studies indicated that STAT3 could be modulated temporarily during virus infection and post-infective dysregulation of immune response; while temporary activation could participate in antiviral response, sustained activation might cause the dysregulation of immune signaling pathways involving the JAK–STAT pathway. Furthermore, complement activation in post-infective autoimmunity is possible, and complement activation is initiated by immune complex formation [40].

The major problem is the size of the main GBS group (GSE31014), which consists of 7 patients and 7 controls. Due to the very low number of observations, the robustness of the model could not be estimated within the GSE31014 group. Hence, a 500-iteration subsampling procedure (7 vs. 7 samples) was performed using the larger T1 vs. HC group. Moderate reproducibility was achieved for a part of the selected genes (DE_freq ~10–33% of total DE genes), all of which have a consistent direction. It is possible that the signal is limited in power but not random. Furthermore, an independent cohort validation analysis was performed using a different human GBS RNA-seq data set (GSE211225; acute vs. control) [41]. After removing the annotations and filters, 28 genes were left from the predefined list of 32-gene shared signature. Out of these genes, 23 genes (82.1%) were consistently upregulated in the independent cohort group. This finding is statistically significant (binomial sign test, p = 9.12 × 10 ⁻ ⁴). In addition, the module had a positive shift overall (log2FC mean = 0.235; log2FC median = 0.258).

On the timeline issue, clinical descriptions of post-infection GBS after flu infection are usually seen at around 5 days to 3 weeks after the exposure, reflecting an early trigger. Nevertheless, immune response amplification leading to neuropathy development may proceed further than the trigger itself. For that reason, we consider weeks 3–4 as an area of interest in terms of investigating the disease mechanism based on its kinetics and latency, but it is not considered a time for intervention [42,43].

Finally, our therapeutic suggestions should be viewed as hypothesis-generating. Molecular docking assesses structural plausibility of interactions but does not demonstrate biological efficacy. Importantly, our study is entirely computational and did not include qPCR, Western blotting, or in vivo/clinical validation; therefore, these results should be interpreted as hypothesis-generating. Concretely, we will pursue a phased validation strategy: (i) *in vitro* assays in H1N1-infected monocytes/macrophages and/or Schwann-cell–relevant systems to test whether candidate compounds attenuate TLR4/TNF/STAT3 signaling and cytokine outputs; (ii) CRISPR/Cas9- or siRNA-mediated perturbation of TLR4 and/or STAT3 under H1N1-mimicking stimulation to assess causality and pathway dependence; (iii) analysis of PBMC/serum from GBS patients with documented recent influenza exposure to evaluate hub-gene expression and inflammatory/complement markers; and (iv) validation in disease-relevant animal models (influenza-infected mice and/or experimental autoimmune neuritis) to test neuroinflammation and therapeutic response in vivo. Future studies using infection-relevant cellular systems, patient-derived samples, and disease models will be required. These studies should test whether modulating the TLR4/STAT3 axis or related immune pathways can reduce post-infectious autoimmune complications.

## Conclusions

This study used integrated transcriptomic and network-based bioinformatic analyses to explore shared neuroimmune signatures between H1N1 infection and GBS. We identified 32 shared differentially expressed genes and found enrichment in immune-regulatory and host–pathogen response pathways. Network analysis prioritized 12 hub genes (including TLR4, ITGAM, and STAT3), highlighting innate immune signaling and inflammatory regulation as potential convergent mechanisms relevant to post-infectious neuropathy.

In addition, in silico drug-repositioning and molecular docking suggested six candidate compounds with plausible binding to selected targets. These predictions provide hypotheses for future experimental testing rather than evidence of therapeutic efficacy.

Given the limited size of the discovery GBS cohort and the computational nature of the analyses, further validation in independent patient cohorts and infection-relevant experimental systems is required, including longitudinal sampling to clarify temporal dynamics and causal relationships. Overall, our results offer a systems-level framework and a prioritized set of genes and candidate compounds to guide mechanistic studies of post-influenza neuroimmune complications.

## Supporting information

S1 Dataset Dataset. (XLSX)
